# Histone Modification Marks Strongly Regulate *CDH1* Promoter in Prostospheres as A Model of Prostate Cancer Stem Like Cells

**DOI:** 10.22074/cellj.2019.5702

**Published:** 2019-02-25

**Authors:** Fatemeh Shokraii, Maryam Moharrami, Nasrin Motamed, Maryam Shahhoseini, Mehdi Totonchi, Vahid Ezzatizadeh, Javad Firouzi, Pardis Khosravani, Marzieh Ebrahimi

**Affiliations:** 1 Department of Developmental Biology, University of Science and Culture, ACECR, Tehran, Iran; 2 Department of Stem Cells and Developmental Biology, Cell Science Research Center, Royan Institute for Stem Cell Biology and Technology, ACECR, Tehran, Iran; 3 School of Biology, College of Science, University of Tehran, Tehran, Iran; 4 Department of Genetics, Reproductive Biomedicine Research Center, Royan Institute for Reproductive Biomedicine, ACECR, Tehran, Iran; 5 Department of Medical Genetics, Royesh Medical Laboratory Centre, Tehran, Iran

**Keywords:** Cancer Stem Cells, *CDH1*, Histone Modification, Methylation, Prostate Cancer

## Abstract

**Objective:**

Cadherin-1 (CDH1) plays an important role in the metastasis, while expression of this protein is under control of epigenetic changes on its gene promoter. Therefore we evaluated both DNA methylation (DNAmet) and histone modification marks of *CDH1* in prostate cancer stem like cells (PCSLCs).

**Materials and Methods:**

In this experimental study, we isolated PCSLCs using cell surface marker and prostaspheroid formation, respectively. The cells isolated from both methods were characterized and then the levels of H3K4me2, H3K27me3, H3K9me2/3 and H3K9ac as well as DNAmet were assessed in *CDH1* promoter of the isolated cells.

**Results:**

The CD44^+^ CD49^hi^ cells were not validated as PCSLCs. However, prostaspheres overexpressed stemness related genes and had higher ability of invasion potential, associated with reduction in *CDH1* expression. Epigenetic status analysis showed that *CDH1* promoter was hypo-methylated. Histone modifications of H3K9ac and H3K4me3 were significantly reduced, in parallel with an increased level of H3K27me3.

**Conclusion:**

Our results suggest that slight decrease of DNAmet of the CpG island in *CDH1* promoter does not significantly contribute to the change of *CDH1* expression. Therefore, histone modifications are responsible in repressing *CDH1* in PCSLCs.

## Introduction

Great advances in basic cancer research have demonstrated presence of the rare cell population (1-2%) with the ability of self-renewal, multi-potency, tumor initiation, tumor growth/ re-growth, drug resistance and metastasis (1, 2). These cells, named tumor initiating cells or cancer stem cells (CSCs), could generally be identified based on the expression of a variety of cell surface markers such as CD24, CD44, CD133, CD166, Trop-1 and EpCAM (3, 4). They are able to form spheres or colonies in defined cultures (3, 5) as well as efflux of certain DNA dyes (6). Several studies have reported that prostate cancer arises from normal epithelial tissue based on genetic changes and chromosomal abnormalities, both of which are responsible for cell transformation, tumor initiation and progression (7). In addition, recent studies have indicated the crucial role of epigenetic regulatory elements in etiology of prostate cancer (8). In this regard, DNA methylation (DNAmet) and post-translational modifications of histones play pivotal role in regulating gene expression and chromatin remodeling (9) involved in tumor initiation and progression (8). Epigenetic alterations could aberrantly render repression or expression of particular genes involved in malignancy, facilitating carcinogenesis and/or human cancers progression. Thus, disruption of either of these processes is strongly observed in almost all human malignancies, including prostate, breast, ovarian, pancreatic and esophageal cancers (8, 10, 11).

It has been demonstrated that alteration of DNAmet as well as histone modification status, in prostate cancer, influences an extensive number of genes involved in cell migration, polarity and metastasis (11, 12). Cluster of differentiation H1 gene cadherin 1 (*CDH1*), as a hallmark of epithelialmesenchymal transition (EMT) event, is mostly repressed by various epigenetic mechanisms. This phenomenon causes a shift from epithelial to mesenchymal phenotype in tumor cells with high potential of invasion and metastasis (13). Thus far, several studies have been performed on the epigenetic status of particular EMT involved genes, including *CDH1* in prostate cancer cell lines, patients’ sample tissues and prostate cancer stem cells (PCSCs) individually (14). However, most of them just focused in one aspect of epigenetic regulation; DNAmet or histone modifications. Therefore, more studies are needed to better understand the effect of both DNAmet and histone modifications in *CDH1* gene, as an important factor for EMT, in PCSCs or prostate cancer stem like cells (PCSLCs).

In the present study, we enriched the PCSLCs from prostate cancer cell lines using two different methods: particular cell surface markers as well as sphere formation. After characterization of PCSLCs and confirmation of the potency of invasion in PCSLCs, level of DNAmet as well as some remarkable histone modification marks was assessed in *CDH1* promoter region. 

## Materials and Methods

### Cell culture

Two human prostate cancer cell lines “prostate stem cell carcinoma (PC3), and human prostate adenocarcinoma cells (LNCaP)” were obtained from National Cell Bank of Iran (NCBI), Pasture Institute, Tehran, Iran. Roswell Park Memorial Institute 1640 (RPMI 1640) and Dulbecco’s Modified Eagle Medium (DMEM, both purchased from Gibco, Germany) were used to culture human prostate cell lines. Both media were supplemented with 2 mM glutamine (Gibco, Germany), 100 U/mL of penicillin and 100 µg/mL streptomycin (Gibco, Germany) and 10% fetal bovine serum (FBS, Gibco, Germany). The cells were preserved in 5% CO_2_ humidified air and 37°C cell culture incubator.

For sphere culture, 105 cells were plated in T25 flask coated with 12 mg/mL of 2-hydroxyethyl methacrylate (poly-HEMA, Sigma, USA) in 95% ethanol, while the flasks were washed once with phosphate buffer saline (PBS) before cell seeding. The cells were cultured in serum-free medium supplemented with 20 ng/mL epidermal growth factor (EGF) and basic fibroblast growth factor (bFGF, both from Royan Biotech, Iran) for four days. Next, prostate spheres were enzymatically dissociated by Trypsin-EDTA (Invitrogen, USA) and maintained at -70°C for future molecular assessments.

### Flowcytometry and cell sorting 

Expression of some stem cell related markers, including CD133, CD44, CD49b, CD29 and CD24 (Table S1) (See Supplementary Online Information at www.celljournal. org), were assessed using BD FACS Aria II (Beckman Dikenson, USA) on the indicated prostate cancer cell lines. To minimize non-specific binding, single cell suspensions were treated with blocking solution before staining (30 minutes on ice). To sort the cells, about 5×106 LNCaP or PC3 cells were stained and sorted in RPMI-1640 medium containing 30% FBS. Post-sorting analysis was performed to ensure the purity of sorted sub-populations.

### Cell doubling time assessment 

PC3, LNCaP and isolated sub-populations were seeded at the concentration of 3×10^3^ cells/well in the 12-well plates. Quantity of the cells was subsequently counted after 72, 120 and 168 hours. Doubling time was calculated based on "(T2-T1)/3.32×(log n2-log n1)", where T2 is the harvesting time; T1 is seeding time; n2 is the number at harvesting and n1 is the number at seeding time.

### Colony formation assay

Briefly, 40 cells of different groups were seeded in each well of 6-well plates. After two weeks culture in the complete RPMI-1640 medium supplemented with 2 mM glutamine (Gibco, Germany), 100 U/mL of penicillin and 100 µg/mL streptomycin (Gibco, Germany) and 10% FBS, number of colonies was counted under the phase- contrast microscope. 

### Spheroid formation assay

5×10^3^ cells/well from prostate cancer cell lines and sorted cells were seeded into 6-well ultra-low attachment plates, in serum-free media supplemented with 20 ng/mL EGF and bFGF. The sphere quantity was subsequently counted after 14 days of growth, using phase contrast microscope.

### Quantitative reverse transcription polymerase chain reaction analysis

The expression of stemness related genes (*OCT4, SOX2, NANOG, c-MYC* and *KLF4*) as well as metastasis related genes (*CDH1* and *CDH2*) were assessed by quantitative reverse transcription polymerase chain reaction (qRT- PCR) in the sorted sub-populations, spheres and parental cells. Briefly, total mRNA was extracted from 2×105 cells with RNeasy Mini Kit (Qiagen, Germany) according to the manufacturer’s instruction. Next, 1 µg of total RNA was reverse transcribed by RevertAid™ H Minus First Strand cDNA Synthesis Kit (Fermentase, USA). Relative qRT-PCR was performed applying cDNA, Power SYBR Green mastermix (Applied Biosystems, USA) and related primers in a 7500 Real-Time PCR System (Applied Biosystems, USA). Glyceraldehyde3- phosphatedehydrogenase (*GAPDH*) specific primers were applied as internal control, in this experiment. The sequences of forward and reverse primers as well as annealing temperatures are listed in Table S2 (See Supplementary Online Information at www.celljournal. org). qRT-PCR was performed triplicate for each biological experiment (n=3). Relative quantification levels were evaluated by 2^-ΔΔCt^.

### Chromatin Immunoprecipitation

Histone modifications of H3K9ac, H3K9me2/3, H3K4me2 and H3K27me3 were analyzed on the regulatory region of *CDH1*, in prostaspheres and parental cells, using chromatin immunoprecipitation quantitative PCR (ChIP-qPCR) technique. In this regards, Orange ChIP kit (Diagenode, Belgium) was used according to the manufacturer’s instruction. Briefly, chromatin derived from 1×10^5^ cells was used for each immunoprecipitation reaction. PCR amplification was performed on the DNA recovered from the ChIP samples as well as the respective total chromatin input by using primers listed in Table S2 (See Supplementary Online Information at www.celljournal.org). Next, immunoprecipitated DNA was quantified by real-time PCR, in a 7500 Real-Time PCR system. The data were expressed as a percentage of input DNA associated with the immunoprecipitated DNA relative to a 1/100 dilution of input chromatin.

### DNA methylation assay

Bisulfite modification of genomic DNA was performed using EpiTecy Bisulfite kit (Qiagen, Germany) according to manufacturer’s protocol. Briefly, a total volume of 140 µL mastermix was made using 1 µg DNA, 35 µL DNA protect buffer and 85 µL bisulfite mixture reagent. The mastermix was respectively incubated at 99°C for 5 minutes, 60°C for 25 minutes, 99°C for 5 minutes, 60°C for 85 minutes, 99°C for 5 minutes and ultimately 60°C for 175 minutes. 

Next, methylation status of 17 CpG sites was evaluated in a 210 bp *CDH1* regulatory region, using the following primers:

F: 5´-TTTTAGGTTAGAGGGTTATT-3´

R: 5´-CTCACAAATACTTTACAATTCC-3´

Bisulfite sequencing PCR (BSP) was performed in a totalvolume of 20 µL, composed of 57-µL of converted DNA, 10pmol of each forward and reverse primers, 1.5 U AmpliTaqGold Polymerase, 10x PCR reaction buffer (containing 15mM MgCl2 and 0.2 mM of each dNTP), using an initialdenaturation at 95°C for 10 minutes, followed by six cyclesof 95°C for 1 minute, 57°C for 1 minute, 72°C for 1 minute and 34 cycles of 95°C for 45 seconds, 53°C for 30 seconds, 72°C for 40 seconds, terminated by incubation at 72°Cfor 10 minutes. The PCR products were analyzed in a 2% agarose gel, and the desired size was purified. The fragmentwas subsequently cloned in Top-10 using InsTA Clone PCRCloning Kit (Thermo Fisher Scientific, USA). 12 positiveclones per sample were selected and prepared for colony- PCR using general M13 primers. The purified products wereultimately sequenced with M13 primers in an ABI 3130-Avantautomated sequencer (Applied Biosystems, USA), followedby alignment and analysis in Chromas (Technelysium Pty Ltd, Australia). 

### Statistical analysis

Data were analyzed by one-way ANOVA using SPSS software. The data are presented as mean ± SD of three different replicates. Athreshold of P<0.05 were considered statistically significant different.

## Results

### Enrichment and characterization of PCSLCs derived from PC3 and LNCap 

**Fig.1:**
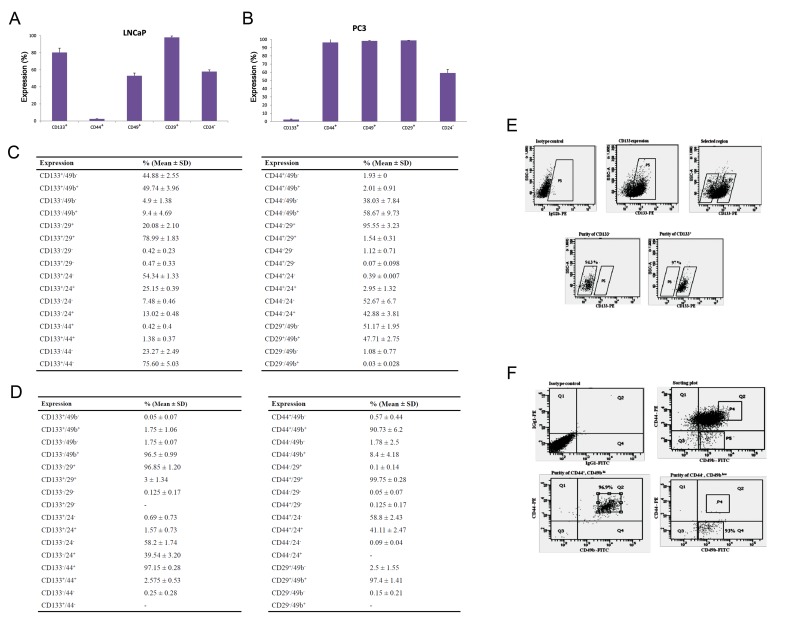
Characterization of prostate cancer stem like cells (PCSLCs). Co-expression of putative stem cell markers in A, C. LNCap and B, D. PC3 prostate cancer lines. Putative cell surface markers for PCSCs were quantified by immuno-fluorescent cell analysis, using FACS machine, E. LNCap cells were sorted based on the expression of CD133 and categorized in two CD133+ and CD133-sub-populations, F. PC3 cells were sorted according to co-expression of CD44 and CD49b, led to classification of them in two different groups: CD44^+^/CD49b^high^ and CD44/CD49blow .

To enrich cancer stem-like cells, protein expression of the identified markers for PCSCs "CD44, CD133, CD29, CD49b and CD24" were firstly examined by FACS in both PC3 and LNCaP cell lines. We found that 80% of LNCaP cells expressed CD133, while only 3% of them were positivefor CD44 (Fig.1A). In contrast, almost all (about 100%) ofthe PC3 cells were positive for CD44 as well as CD49b, and only about 3% of them expressed CD133 (Fig.1B). Both lines were positive for CD29 and about 60% of thesecells were negative for CD24. With regards to expressionof the aforementioned surface markers (Fig.1C, D), LNCaPwas sorted upon co-expression of CD133 and CD49b infour different groups: CD133+/CD49b+, CD133-/CD49b-, CD133+/CD49b-and CD133-/CD49b-, none of which had difference in doubling time and cell growth (Data are notshown). They were subsequently sorted only according toexpression of CD133 (Fig.1E). PC3 cells were also sortedbased on co-expression of CD44 and CD49b in two differentgroups: CD44^+^/CD49b high and CD44^-^/CD49^blow^ (Fig.1F). Purity of the isolated populations was generally more than 90% in each group.

**Fig.2:**
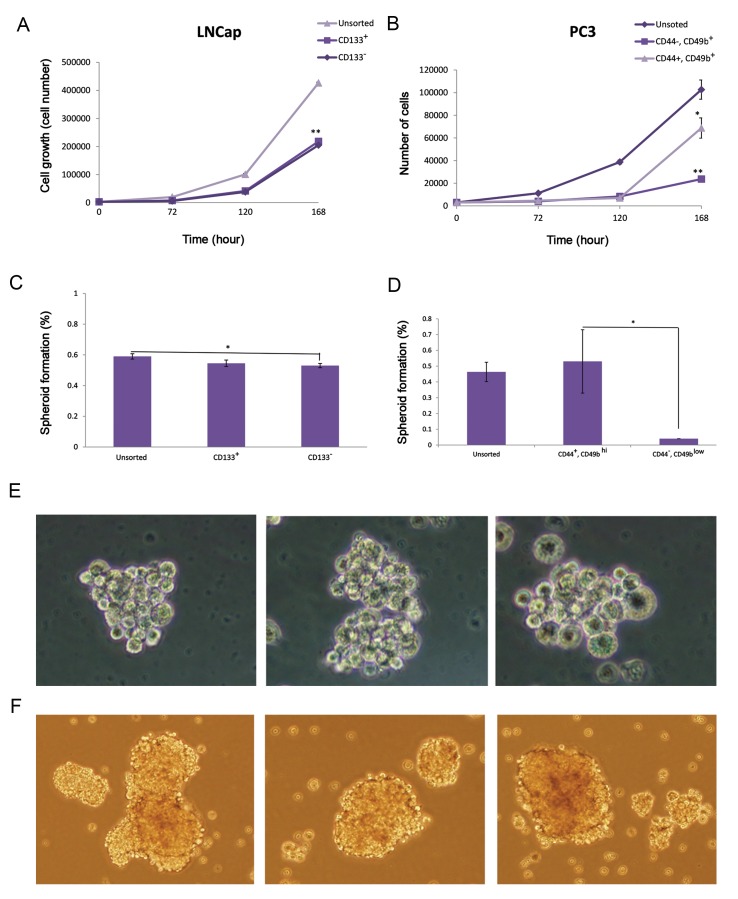
Characterization of the sorted cancer cells based on proliferation rate and ability to form spheres. Cell viability was assessed in A. CD133+ and CD133-LNCaP cells, B. CD44^+^/CD49b^high^ and CD44^-^/CD49^blow^ PC3 cells, compared to unsorted cells by MTT assay during 168 hours of culture. Sphere formation capacity were evaluated on serum free medium supplemented with basic fibroblast growth factor (bFGF), epidermal growth factor (EGF) and B27 in low attach culture dishes in C. LNCaP sorted cells, D. PC3 cells (n=3, *; P=0.05, **; P=0.01). Morphology of spheroids derived from E. LNCaP (scale bar: 50 µm), and F. PC3 sorted cells and parental cells (scale bar: 50 µm).

To characterize the sorted cells, obtained from LNCaP and PC3, cell growth as well as colony (under diluted conditions) and spheroid (in serum-free medium under low attachment culture conditions) formation capacities were tested. Our findings demonstrated no significant difference in the cell growth and sphere formation ability in unsorted, CD133+ and CD133-cells isolated from LNcap (doubling time: 22.6, 22.37 and 22.09 hours, respectively, P≥0.05, Fig.2A, C). However, these abilities were higher in CD44^+^/CD49b^high^ and PC3 unsorted cells, compared to CD44^-^/CD49^blow^ (doubling time in unsorted and CD44^+^/CD49b^high^ cells was 32.025 and 29.685 hours, respectively, P<0.004). Meanwhile, unsorted cells and CD44^+^/CD49b^high^ showed approximately 12.6 ± 1.1 fold increase in spheroid formation than CD 44-/CD49bDim (P<0.05, Fig.2B, D).

Morphologically spheroids derived from LNCaP were large, round shape and tightly packed (Fig.2E), whereas the PC3 spheroids were grapes-like, loosely packed containing fewer cells (Fig.2F).

**Fig.3:**
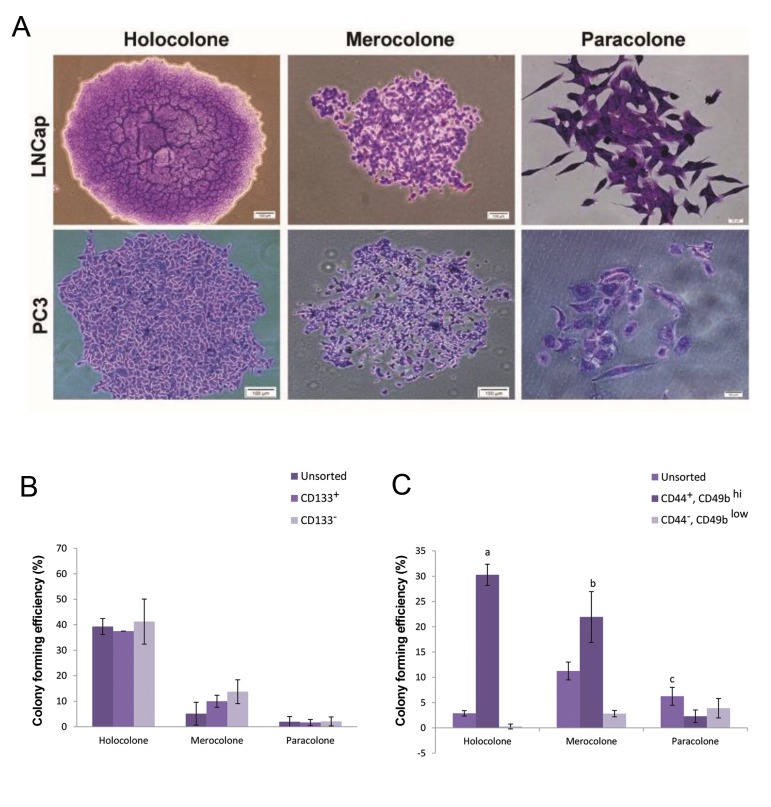
Colony forming efficiency of PC3 and LNCaP sorted cells. A. Images show phase-contrast micrographs of the stained colonies with crystal violet, B. Graphs represent the frequency of holoclones, meroclones and paraclones in LNCaP, and C. PC3 and their sorted groups after 14 days of culture. The frequency of each type of colonies was determined in three replicate (mean ± SD). a; P=0.001 vs. all, b; P=0.01 vs. all, and c; P=0.05 vs. CD44^+^/ CD49b^high^.

The results of colony formation assay revealed that LNCaP, PC3 and their relevant sorted groups yielded a mixture ofcolony morphologies after 6-7 days of culture, classified asholoclones, meroclones and paraclones. The holoclones wereround shape and large in size with tightly packed small cells, whereas paraclones had irregular shape and comprised of loosely packed flattened and scattered cells. Meroclones were intermediate in terms of the size and number, while they were mixture of holoclones and paraclones (Fig.3A). There was no significant difference in colony forming efficiency between unsorted, CD133+ and CD133-LNCaP cells (Fig.3B). In PC3, the CD44^+^/CD49b^high^ cells were more capable to form holoclones (30.3%) and meroclones (21.95%), compared to CD44^-^/CD49^blow^ (with a respective rate of 0.27 and 2.81%) as well as unsorted PC3 cells with a range of 2.87 and 11.25%, respectively (Fig.3C).

Taken together, these data demonstrated that CD133 was not specific marker for identification of PCSCs in LNCaP line. While the PC3 CD44^+^/CD49b^high^ sub-population revealed cancer stem-like properties. Therefore, we selected PC3 cells for further analysis from molecular aspect.

### Stemness and EMT related gene expressions in CD44^+^/ CD49b^high^ and prostaspheres 

**Fig.4:**
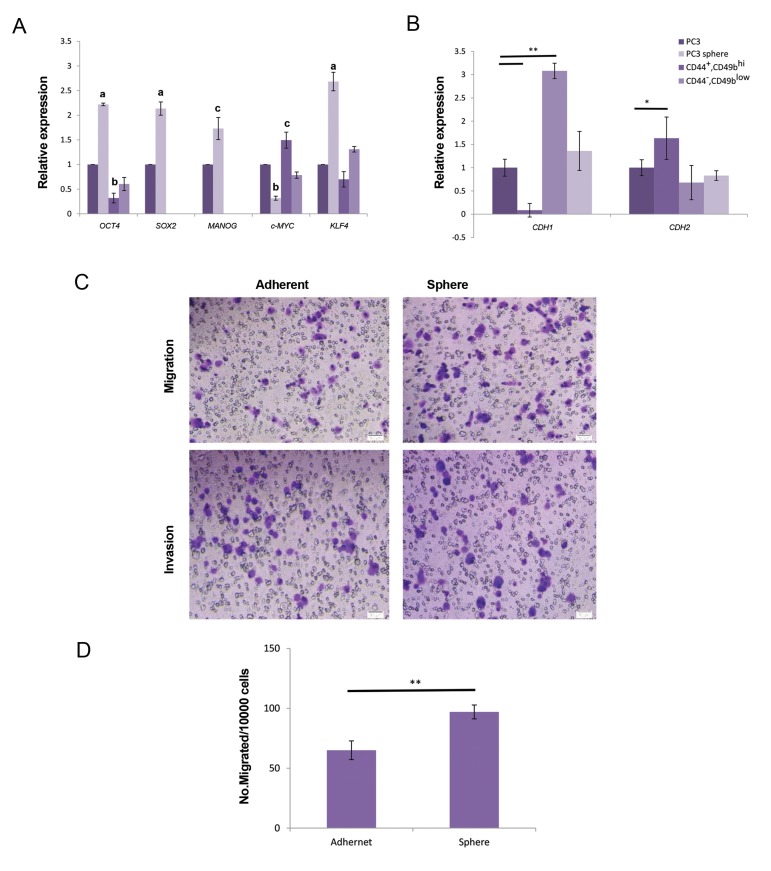
PC3 cancer stem cells (CSC) characterization. A. The expression level of stemness related genes and B. Important metastasis related genes were assessed by quantitative reverse transcription polymerase chain reaction (qRT-PCR) in PC3 and the respective sub-populations. Expression levels were normalized to Glyceraldehyde-3-phosphatedehydrogenase (*GAPDH*). a; P≤0.001 vs. all, b; P≤0.01 vs. all, c; P≤0.05 vs. PC3, **; P≤0.01, and *; P≤0.05, C. Morphology of the migrated and invaded cells. Quantification of D. Migrated, and E. Invaded cells (n=3, mean ± SD, **; P≤0.01).

The expression of *OCT4, SOX2, NANOG, c-MYC, KLF4,* as stemness related genes, as well as *CDH1* and *CDH2*, as EMT related genes, were analyzed in the sorted cells and prostaspheres derived from PC3. All stemness related genes except c-MYC were significantly over- expressed in prostaspheres (Fig.4A, P<0.05). Conversely, expression of c-MYC was significantly down-regulated (P=0.007). Most of the stemness related genes were not increased or even contrarily were down-regulated in both sorted cells obtained from PC3 cells (Fig.4A). *CDH1* was strongly down-regulated in PC3-prostaspheres, while their invasion and migration potentials were significantly increased (Fig.4B-E). Interestingly, *CDH1* was over- expressed in CD44^+^/CD49b^high^, while the respective change was not significant in the counterpart sorted cells (Fig.4B). The changes in N-cadherin gene (*CDH2*) were not significant in neither groups (Fig.4B, P>0.05). 

### Differential DNA methylation and histone B modifications of the *CDH1* promoter in prostaspheres and parental cells

**Fig.5:**
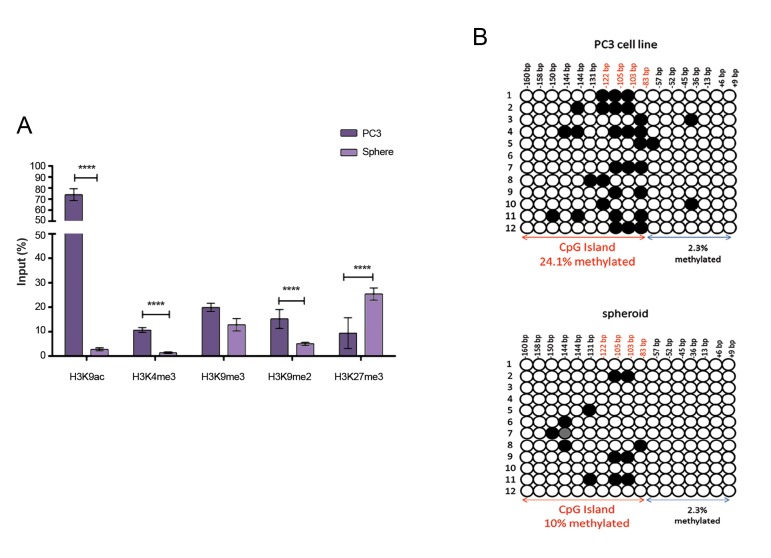
Epigenetic characterization of PC3 cancer stem cells (CSC). A. ChIP analysis of histone modifications on the regulatory region of *CDH1* in PC3 spheroids and the respective parental cells. The results are expressed according to a 1/100 dilution of input chromatin and B. DNA methylation level of *CDH1* promoter in the PC3 spheroids andits parental cells. Polymerase chain reaction (PCR) was carried out for a CpG island within the promoter. All of the epigenetic data are reported as the mean ± SD (n=3), ****; P<0.0001, and ***; P<0.001.

The main purpose of this study was to understand the epigenetic alterations of H3K9ac, H3K9me2/3, H3K4me3 and H3K27me3, as well as DNAmet of the *CDH1* regulatory region in the PCSLCs. A significant hyper-acetylation of lysine 9 of histone 3 was observed in the *CDH1* promoter of PC3 cells (Fig.5A). However, this was reduced 40 fold in PC3 spheres. In addition, methylation of histone 3 at lysine 4, representing an open and euchromatin form of the *CDH1* regulatory region, was reduced 10 fold in the PC3 spheres. In contrast, the repressive mark of H3K27me3 was increased 2.5 fold in the spheres (P<0.05). Among the repressive markers of H3K9me2/3, H3K9me2 was reduced significantly (P<0.05), but the change of H3k9me3 was not significant in the PC3 spheres. 

The prostaspheres were more hypo-methylated than parental cells, especially in the -83, -103, -105 and -122 bp regions of *CDH1* (Fig.5B, Table 1). Thus, we determined that the latter four CpG regions had maximum effect on the regulation of *CDH1*. 

**Table 1:** Rang of CDH1 promoter CpG methylation (%) in PC3 spheroids, sorted cells and the parental cells


Group	-122 bp	-105 bp	-103 bp	-83 bp

PC3 Cells	33	58	41	58
CD44^-^/CD49b^+^	11	22	22	11
CD44^+^/CD49b^+^	11	22	22	11
Spheroids	0	25	25	8


## Discussion

This is the first study on simultaneous evaluation of histone and DNAmet for the regulatory region of *CDH1* gene in the PCSLCs. In the first step, we attempted to isolate PCSLCs based on the previously reported stem cell surface markers; however, it was not successful. In fact, the CD133+ and CD133-sorted LNCap cells (nonmetastatic line) had no significant difference in terms of colony and spheroid formation abilities. Moreover, isolation of CSCs based on the co-expression of CD44 and CD49b in PC3 cells (metastatic line), resulted in more clonogenic with higher number of spheroids in CD44^+^/ CD49b^high^ cells. Nevertheless, no difference on the ability of colony and sphere formations as well as stemness related gene expressions were determined between CD44^+^/CD49b^high^ and parental cells. 

Although, since 2005, many reports have shown that CD133 alone or in combination with CD44 and/or a2b1 integrin is a stem like population marker and it is used for isolation of PCSCs (15, 16), some experimental models have shown that cells expressing those markers are not appropriately enriched in stem cell population of prostate cancer (17, 18). Therefore, we suggest that determining only cell surface markers could not be an authentic method for isolating CSCs from prostate cancer lines. This is indeed consistent with previous reports in the other cancer types (19). It has been reported that cell surface markers could easily be changed based on cell density, number of passages, pH of the culture medium and length of culture. Thus, in long term passaging some of the cell surface markers are diminished (20). Isolation of CSCs, using cell surface marker, might be favorable in patient’s tumor biopsies or tumor primary cultures. 

The other approach to enrich CSCs from cell lines or tumor biopsies is sphere formation. CSCs are the only cells with an ability of proliferation in serum free culture and non-anchorage dependent way (21). Our preliminary results revealed that PC3-spheres are clonogenic and able to migrate as well as extravasate from matrigel layer *in vitro*.

In advanced human prostate tumors, expression of *CDH1* is strongly reduced. The initial evidences indicated that DNA hyper-methylation could inactivate *CDH1* (22, 23). Curiously, other study later demonstrated that changes in histone modifications, rather than DNAmet, may be the predominant factor in reactivation of *CDH1* expression (23, 24) in cancers. Moreover, genome wide mapping of DNAmet demonstrated that most robust CpG island promoters are unmethylated, even in the gene inactive status. In contrast, low CpG content promoters are largely methylated, while this methylation does not prevent gene expression (25). All of these findings provide that notion of epigenetic regulation complexity based on histone modification and DNAmet is far from what yet understood. Thus far, no study has been performed in the context of both histone modification and DNAmet, in regulatory site of *CDH1* in PCSCs. In line with previous studies, our results demonstrated that upon CSC enrichment, the expression of *CDH1* was reduced and these isolated cells represented more aggressive fate, resulted in higher potential of migration and invasion in vitro. DNAmet analysis showed significant hypo-methylation of *CDH1* promoter CpG site. Among chromatin remodeling factors, reduction in the canonical epigenetic marks of transcription initiation (H3K4me3 and H3K9me3) and enhancement in the repressive mark H3K27me3 was observed in prostaspheres derived from PC3 cells. Although, DNA promoter hyper-methylation was previously reported as the principle epigenetic cause of silencing *CDH1* (22, 26, 27), other studies highlight that H3K27me3 activity plays more important role in *CDH1* repression (28). Considering that DNAmet event generally happens in the repressed gene promoter regions of normal cells, Ke et al. (24) reported low correlation between DNAmet and gene silencing of EMT related genes in prostate cancer. 

In this context, several possible mechanisms might be involved for *CDH1* gene silencing of prostaspheres. Firstly, methylation could gradually be lost over the sphere formation, as CSLCs in culture grow earlier than methylation and it can be copied from the replicating parental DNA, resulting in progressive loss of DNAmet (29-32). Secondly, *CDH1* gene silencing is influenced by both DNAmet and chromatin remodeling factors, with an attention toward the latter factors. Thus, the effect of H3K9ac and H3K4me3, as two activating markers, and H3K27me3, as repressing marker, is stronger than DNA hypo/ hyper-methylation in gene expression. It is proposed that DNAmet is a strong silencing mark, while the genes are modified by only DNA methyl transferases (DNMTs), without concomitant of H3K4me3. In this respect, it appears that DNAmet of promoters could just slightly contribute in gene repression. Thirdly, it is proposed that both DNAmet and H3K4me3 have similar complementary effect on some gene expressions. Consistent with this, several studies have demonstrated that individual activity of DNAmet, in absence of H3K4me3, suppressed gene transcription, while this effect was slightly reduced in combination of DNA and H3K4 methylations (24, 33). In addition, investigations revealed that increased H3K4me3 caused several gene up-regulations during EMT, while they were down-regulated with H3K4me3 reduction. Controversy effect of H3K27me3 is observed in gene regulation; whereby most of the genes with increased H3K27me3 were under-expressed through EMT process, while genes with decreased H3K27me3 were up-regulated. However, no significant correlation was observed between DNAmet and gene expression levels throughout EMT (24). Previously, genome- wide analyses of H3K4me3 and H3K27me3 showed a strong correlation between H3K4me3 gene expression, in addition to the correlation of H3K27me3 activity, and gene repression in embryonic stem cells (34-37), T-cells (38) hematopoietic stem cells/progenitor cells (39) as well as prostate cancer cells (40). Fourthly, it has been hypothesized that bivalent H3K4me3/ K27me3 is a repressive mark and H3K4me3/DNAmet is an activation mark in prostate cells. Interestingly, investigations implicated that the marked genes with H3K4me3/DNAmet are preferentially active (24) which suggests a misleading conclusion in the case of predicting gene expression based only on the activity of DNA methylated without considering H3K4me3 modification.

## Conclusion

These findings indicate the complicated epigenetic regulation of the *CDH1* promoter at prostaspheres, as a model of PCSLCs. In *CDH1* promoter region of these spheres, H3K27me3 was enhanced. In contrast, three histone modification marks (H3K9ac, H3K4me3, H3K9me2) and DNAmet were reduced, despite down- regulation of the respective gene. This finding correlated with enhancement of metastasis potential and accumulation of malignant features. In this model, we suggested that slight decrease of DNAmet of the CpG island in *CDH1* promoter does not significantly contribute to the change of *CDH1* expression. Therefore, histone modifications are responsible in repressing *CDH1* expression in PCSLCs.

## Supplementary PDF


