# Supplementary material for: Histone Modification Marks Strongly Regulate CDH1 Promoter in Prostospheres as A Model of Prostate Cancer Stem Like Cells
Source: Cell J. 2019 Feb 25;21(2):124–34. doi: 10.22074/cellj.2019.5702 (PMC6397603; doi:10.22074/cellj.2019.5702)
Supplement: Supplementary file 1 [file Cell-J-21-124-s01.pdf]

Supplementary Information for

Histone Modification Marks Strongly Regulate *CDH1* Promoter in Prostospheres as A Model of Prostate Cancer Stem Like Cells

Fatemeh Shokraii, M.Sc.[1, 2#], Maryam Moharrami, M.Sc.[3#], Nasrin Motamed, Ph.D.[3*], Maryam Shahhoseini, Ph.D.[4], Mehdi Totonchi, Ph.D.[4], Vahid Ezzatizadeh, Ph.D.[2, 5], Javad Firouzi, M.Sc.[2], Pardis Khosravani, M.Sc.[2], Marzieh Ebrahimi, Ph.D.[2*]

1. Department of Developmental Biology, University of Science and Culture, ACECR, Tehran, Iran
2. Department of Stem Cells and Developmental Biology, Cell Science Research Center, Royan Institute for Stem Cell Biology and Technology, ACECR, Tehran, Iran
3. School of Biology, College of Science, University of Tehran, Tehran, Iran
4. Department of Genetics, Reproductive Biomedicine Research Center, Royan Institute for Reproductive Biomedicine, ACECR, Tehran, Iran
5. Department of Medical Genetics, Royesh Medical Laboratory Centre, Tehran, Iran

#The first two authors equally contributed to this work.

**Corresponding Addresses: P.O.Box: 14155-6455, School of Biology, College of Science, University of Tehran, Tehran, Iran*
P.O.Box: 16635-148, Department of Stem Cells and Developmental Biology, Cell Science Research Center, Royan Institute for Stem Cell Biology and Technology, ACECR, Tehran, Iran
Emails: motamed2@khayam.ut.ac.ir, mebrahimi@royaninstitute.org

Table S1: List of antibodies and isotype controls

Antibody name/Isotype name	Isotype	Cat. No.	Company
APC Mouse Anti-Human CD29	IgG1	559883	BD
PE Mouse Anti-Human CD29	IgG1		BD
FITC Mouse Anti-Human CD49b	IgG1	555498	BD
PerCP-Cy™5.5 Mouse Anti-Human CD44	IgG2b	560531	BD
FITC Mouse Anti-Human CD44	IgG2b	555478	BD
PE Mouse Anti-Human CD44	IgG1	555479	BD
PE Mouse Anti-Human CD133	IgG2b	130-090-853	Miltenyi Biotec
FITC Mouse Anti-Human CD24	IgG2a	555427	BD
APC Mouse IgG1	-	555751	BD
PE Mouse IgG1	-	12-4714	e-Bioscience
FITC Mouse IgG1	-	12-4714-73	e-Bioscience
PerCP-Cy™5.5 Mouse IgG2b	-	558304	BD
PE Mouse IgG2b	-	X0951	DAKO
FITC Mouse IgG2b	-	X6941	DAKO
PE Mouse IgG2a	-	X0951	DAKO
FITC Mouse IgG2a	-	X0933	DAKO

Table S2: List of the primer sequences

Gene	Primer sequence (5′-3′)	Tm (°C)
GAPDH	F: CTCATTTCCTGGTATGACAACGA<br>R: CTTCCTCTTGTGCTCTTGCT	60
OCT4	F: GTTCTTCATTCACTAAGGAAGG<br>R: CAAGAGCATCATTGAACTTCAC	60
SOX2	F: GGGAAATGGAAGGGGTGCAAAAGAGG<br>R: TTGCGTGAGTGTGGATGGGATTGGTG	60
KLF4	F: ATTACCAAGAGCTCATGCCA<br>R: CCTTGAGATGGGACTCTTTG	60
NANOG	F: AAAGAATCTTCACCTATGCC<br>R: GAAGGAAGAGGAGAGACAGT	60
c-MYC	F: ACACATCAGCACAACTACG<br>R: CGCCTCTTGACATTCTCC	60
CDH1	F: CAGGAGTCATCAGTGTGGT<br>R: GGAGGATTATCGTTGGTGTCAG	60
CDH2	F:5´ GCCCAAGACAAAGAGACCC 3´<br>R: CTGCTGACTCCTTCACTGAC	60.5
CDH1 (regulatory region)	F: TCCCATAACCCACCTAGACC<br>R: TTCCGACGCCACTGAGAG	62
